# Fluid resuscitation practice patterns in intensive care units of the USA: a cross-sectional survey of critical care physicians

**DOI:** 10.1186/s13741-016-0035-2

**Published:** 2016-06-16

**Authors:** Timothy E. Miller, Martin Bunke, Paul Nisbet, Charles S. Brudney

**Affiliations:** Department of Anesthesiology, Duke University Medical Center, Durham, NC 27710 USA; Department of Medical Affairs, Grifols, 79 TW Alexander Dr. Bldg. 4101, Research Triangle Park, NC 27709 USA; One Research, LLC, 1150 Hungry Neck Blvd., Suite C-303, Charleston, SC 29464 USA

**Keywords:** Fluid resuscitation, Intensive care unit, Sepsis, Bleeding, Colloids, Crystalloids, Albumin, Survey

## Abstract

**Background:**

Fluid resuscitation is a cornerstone of intensive care treatment, yet there is a lack of agreement on how various types of fluids should be used in critically ill patients with different disease states. Therefore, our goal was to investigate the practice patterns of fluid utilization for resuscitation of adult patients in intensive care units (ICUs) within the USA.

**Methods:**

We conducted a cross-sectional online survey of 502 physicians practicing in medical and surgical ICUs. Survey questions were designed to assess clinical decision-making processes for 3 types of patients who need volume expansion: (1) not bleeding and not septic, (2) bleeding but not septic, (3) requiring resuscitation for sepsis. First-choice fluid used in fluid boluses for these 3 patient types was requested from the respondents. Descriptive statistics were performed using a Kruskal-Wallis test to evaluate differences among the physician groups. Follow-up tests, including *t* tests, were conducted to evaluate differences between ICU types, hospital settings, and bolus volume.

**Results:**

Fluid resuscitation varied with respect to preferences for the factors to determine volume status and preferences for fluid types. The 3 most frequently preferred volume indicators were blood pressure, urine output, and central venous pressure. Regardless of the patient type, the most preferred fluid type was crystalloid, followed by 5 % albumin and then 6 % hydroxyethyl starches (HES) 450/0.70 and 6 % HES 600/0.75. Surprisingly, up to 10 % of physicians still chose HES as the first choice of fluid for resuscitation in sepsis. The clinical specialty and the practice setting of the treating physicians also influenced fluid choices.

**Conclusions:**

Practice patterns of fluid resuscitation varied in the USA, depending on patient characteristics, clinical specialties, and practice settings of the treating physicians.

**Electronic supplementary material:**

The online version of this article (doi:10.1186/s13741-016-0035-2) contains supplementary material, which is available to authorized users.

## Background

Fluid resuscitation is a cornerstone of intensive care treatments, and fluid therapy is one part of a complex strategy in hemodynamic resuscitation (Myburgh and Mythen 2013). There is considerable debate about the effects of fluid type, timing of administration, appropriate amount of fluid, and techniques for determining fluid responsiveness (Cherpanath et al. 2014; van Haren and Zacharowski 2014). The principles of fluid exchange and how they influence clinical decisions regarding fluid type have been a major topic of interest. With approximately 1.7 million inpatient stays associated with sepsis during 2009, sepsis has become the sixth most common reason for hospitalization in the USA (Elixhauser et al. 2006). Thus, it is of interest to understand how physicians approach fluid resuscitation in sepsis relative to nonseptic conditions. Since 1896, Starling’s Principle of Fluid Exchange stated that fluid movement across the capillary wall depends on the balance between the hydrostatic pressure gradient that pushes water outward into the interstitial space and the colloid oncotic pressure that pulls water inward into the vessel (Aditianingsih and George 2014). As we come to better understand that successful fluid resuscitation depends on the disease “context” (i.e., the physiological state of the patient), it becomes apparent that the classic Starling’s Principle does not apply to all situations and needs to be adapted to encompass conditions involving systemic inflammation and vascular barrier damage (Jacob and Chappell 2013).

In recent years, new research efforts increasingly recognize the endothelial glycocalyx layer (EGL) as a crucial determinant of vascular barrier function (Weinbaum et al. 2007; Becker et al. 2010). The EGL acts as a filter that generates an effective colloid oncotic pressure with the presence of a protein-free layer in the subglycocalyx space of the EGL. Large molecules, (e.g., albumin in colloid solutions) are retained inside the vessel, generating colloid oncotic pressure in the intravascular compartment (Myburgh and Mythen 2013; Aditianingsih and George 2014; Jacob and Chappell 2013). Small molecules (e.g., electrolytes in crystalloid solutions) traveling freely through the vessel wall can draw water into the interstitial space. Animal and human studies suggest that the EGL is damaged in numerous systemic inflammatory states, including trauma (Johansson et al. 2011) and sepsis (Steppan et al. 2011), and may become compromised, leading to interstitial edema (Weinbaum et al. 2007; Ait-Oufella et al. 2010). In other words, colloids may behave more like crystalloids in sepsis, and several large studies have failed to show any benefit from colloids in this context. On the other hand, if a patient has an intact EGL and an intravascular volume deficit, then volume therapy with a colloid fluid restores the intravascular volume as predicted by Starling, and far higher volumes of crystalloids are required to achieve the same result (Rehm et al. 2000). In this context, at least theoretically, colloids may have advantages over crystalloids (Roger et al. 2014).

In addition, there is still on-going debate over whether liberal or restricted fluid volume strategies would yield more favorable clinical outcomes in critically ill patients (Polderman and Varon 2015). What is generally accepted is that fluid administration should be managed to achieve zero or negative fluid balance by the time patients recover from all 4 phases of fluid resuscitation [(1) salvage/rescue, (2) optimization, (3) stabilization, (4) de-escalation] (Vincent and De Backer 2013; Myburgh 2015; Hoste et al. 2014; Rewa and Bagshaw 2015). The context of where the patients are in their course of critical illness is important (Vincent and De Backer 2013). The FIRST (James et al. 2011) and CRISTAL (Annane et al. 2013) trials enrolled patients undergoing resuscitation for highly severe trauma and severe hypotensive, hypovolemic shock, respectively, which presumably mean those patients were in the *salvage/rescue phase*. On the other hand, the SAFE (Finfer et al. 2004), CHEST (Myburgh et al. 2012), and ALBIOS (Caironi et al. 2014) trials enrolled patients who were mostly in the *optimization phase* with lower fluid volume needs and likely longer time interval from shock onset. Likewise, other trials (Navarro et al. 2015; Opperer et al. 2015) conducted in the perioperative disease context were likely in the *optimization phase*.

It is important to assess how fluids are currently being used in the USA for sepsis and other critical care conditions. The objectives of this study were the following: (A) to examine the use of different types of fluids for resuscitation (i.e., crystalloids, plasma-derived colloid [albumin], synthetic colloids [hydroxyethyl starches, HES]) in critically ill patients in adult intensive care units within the USA; (B) to determine whether certain patient characteristics and/or practice settings have an influence on the type of fluid utilized for resuscitation; and (C) to determine whether the fluid selected for resuscitation varies by clinical specialties of the treating physicians.

## Methods

### Study design

This study is cross-sectional and collected survey data from physicians practicing in medical and surgical ICUs of the USA. A 10-min online survey was administered to 502 physicians to investigate the patterns of fluid utilization in the ICU. Initial survey questions were developed by 2 of the authors (TM and CSB), finalized through discussion and input from all authors then pretested to ensure the quality of the survey. The 25-item self-administered questionnaire (Additional file 1) obtained information on preferences for fluid use in hemodynamic management and volume status indicators used most often to determine volume expansion needs. The survey questions were designed to assess clinical decision-making for 3 types of patients: “patient type 1” needs volume expansion but is not bleeding and not septic, “patient type 2” needs volume expansion in the presence of blood loss when blood transfusion is not indicated (adequate Hb) and the patient is not septic, and “patient type 3” needs volume expansion for resuscitation in sepsis. First-choice fluid used in fluid boluses for these 3 different types of patients was requested from the respondents. In addition, physicians were presented with the 3 patient scenarios sequentially and asked to identify their first choice of fluid from a list of 5 colloid and crystalloid solutions for volume expansion for each patient type. The 5 types of fluids were crystalloids, 5 % albumin, 25 % albumin, 6 % HES 450/0.70 and 6 % HES 600/0.75 (first-generation HES), and 6 % HES 130/0.4 (third-generation HES). For simplification, we will refer to “6 % HES 450/0.70 and 6 % HES 600/0.75” as *HES 450/600* and “6 % HES 130/0.40” as *HES 130* throughout the rest of this manuscript. Physicians rated the frequency with which they preferred various products for volume expansion using a 5-point scale, from “always” to “never,” and they were also asked to indicate the bolus volume (milliliter) of the crystalloids and colloids that they typically use for volume expansion. In addition, they were asked to rate the importance of certain colloid characteristics (e.g., more sustained volume expansion, faster volume expansion) and nononcotic properties of albumin (e.g., transport of metabolites, free radical scavenging) on the treatment decision-making process using a 5-point scale, from “not important” to “absolutely essential.”

In February 2015, participants were recruited from the Research Now Healthcare physician panel. Research Now is a company that manages a panel of physicians who have opted to become members of the Research Now Healthcare panel. Email invitations for participation in this study were sent from Research Now to their physician panelists, who remained anonymous to the investigators in this study. To qualify to participate in this survey, physicians had to specialize in anesthesiology, surgery, critical care medicine, or pulmonology; have been in practice for at least 2 years since residency; rotate in surgical ICU, medical ICU, or an ICU accepting a variety of patients; and treat or consult on at least 3 to 4 ICU patients per week. Physicians who worked in cardiac, neurology, or pediatric ICUs were excluded. Subquotas were set for each specialty to ensure that a minimum number of completed surveys were received from each specialty: 125 surgeons, 125 anesthesiologists, 175 critical care medicine specialists, and 75 pulmonologists. All potential respondents were recruited by email invitation which provided a general description of the survey topic and a link for interested recipients to access the online survey. Invitations were sent to 12,435 physicians. Of these, a total of 2724 physicians attempted to access the survey, making the response rate 21.9 %. Of the 2724 who attempted to access the survey, 502 (4.0 %) made up the final participant pool that accessed the survey, qualified via the screening questions, and completed the survey prior to the set quotas being reached. Each invitation contained a unique ID that prevented any one respondent from taking the survey more than once. The participants were aware that the anonymous data collected in this survey may be published.

This research project involved obtaining the opinions of physicians about their choice for the use of various fluids for resuscitation in 3 different hypothetical patient situations. No patient data was obtained, and no questions were asked of the physicians that would help in identifying them. Any physician data was de-identified. Hence, this study was exempt from requiring institutional review board review under USA Code of Federal Regulations Title 45 Part 46.101(b)(2) by Copernicus Group IRB, because any physician information within the survey dataset was de-identified.

### Statistical analysis

Several questions were based on 5-point scales and provided ordinal data which, by definition, are not normally distributed. As such, descriptive statistics were performed using a Kruskal-Wallis test to evaluate differences among the physician groups on the ordinal measures. Follow-up tests were conducted to evaluate differences between ICU types and between practice settings. *t* tests were used to evaluate differences across ratio variables (i.e., bolus volume). Statistical significance was assessed at the alpha level of <0.05. Descriptive analyses were performed using SPSS (version 23.0). Data analysis was done by PN.

## Results

Of the 502 physicians who completed the survey, 125 (24.9 %) were anesthesiologists, 125 (24.9 %) were surgeons, 104 (20.7 %) practiced critical care medicine, and 148 (29.5 %) were pulmonologists (Table 1). The majority of anesthesiologists and surgeons practiced in surgical ICUs, while the majority of critical care medicine specialists and pulmonologists were from medical ICUs. Approximately three-fourths of the physicians from each clinical specialty practiced at nonuniversity hospitals. The average hospital size was approximately 400 beds, and the average number of ICU beds in each hospital was about 40 beds. Physician age and gender were not collected.

**Table 1:** Summary of participant characteristics

		Anesthesiologists^A^	Surgeon^S^	Critical care medicine^C^	Pulmonologists^P^
		(*n* = 125)	(*n* = 125)	(*n* = 104)	(*n* = 148)
ICU type	Surgical	58.4 %	74.4 %^A^	–	–
	Medical	–	–	52.9 %	61.5 %
	ICU accepting variety of patients	41.6 %^S^	25.6 %	47.1 %^S^	38.5 %^S^
Practice setting	University hospital	24.8 %	28.0 %	31.7 %	26.4 %
	Nonuniversity hospital	75.2 %	72.0 %	68.3 %	73.6 %
Hospital size	No. of beds in the hospital	343	410	410	403
	No. of ICU beds in the hospital	35	49^A^	39	41

*Superscripts A*, *S*, *C*, and *P* denote differences between specialties that are statistically significant at *P* < 0.05

*ICU* intensive care unit

**Fig. 1:**
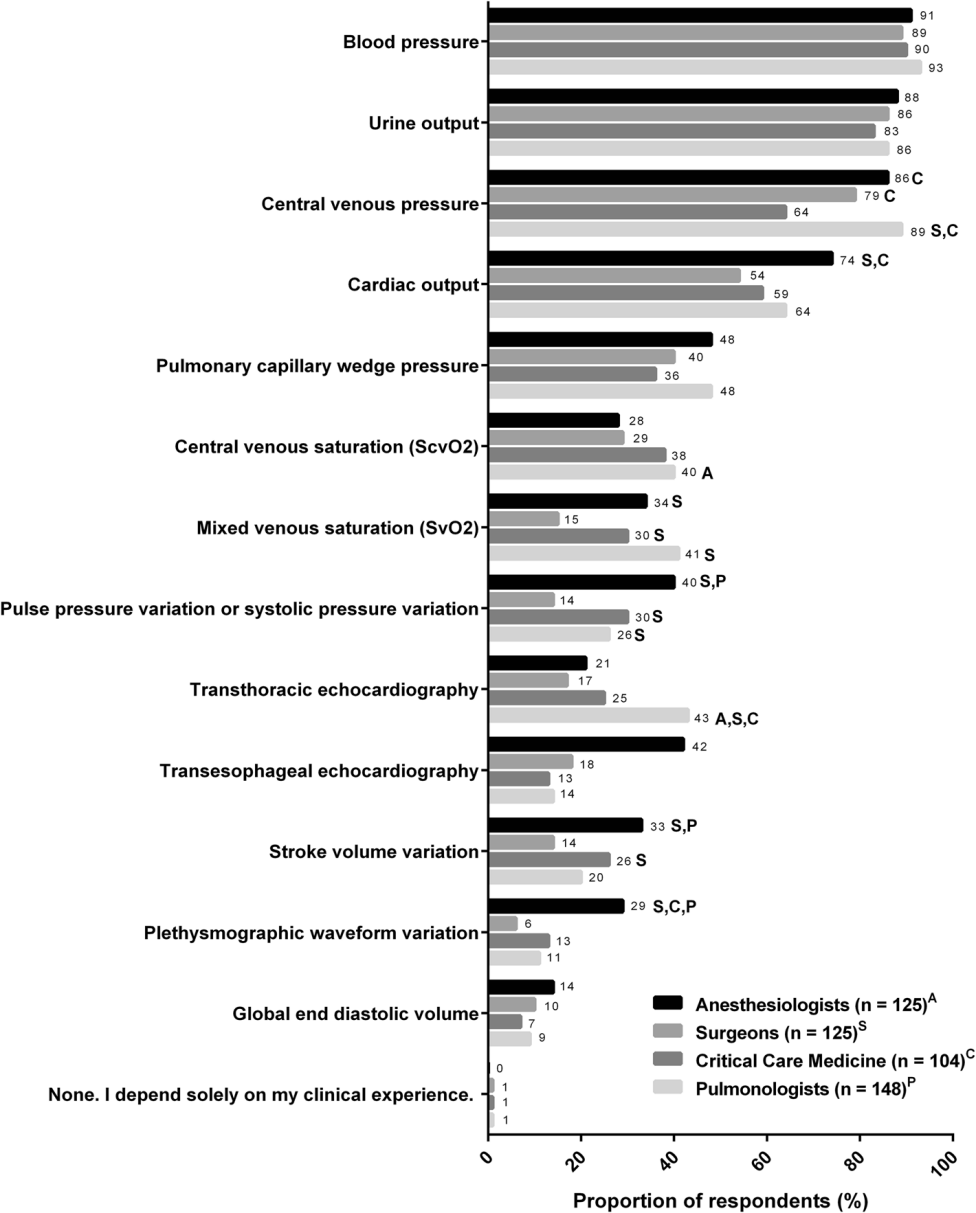
Volume status indicators and diagnostic tools used in assessing fluid needs. *Superscripts A*, *S*, *C*, and *P* denote differences between specialties that are statistically significant at *P* < 0.05

The decision-making process for fluid management varied considerably among physicians of all clinical specialties in this study, and there was extensive heterogeneity in the diagnostic approaches used to inform decisions for fluid management (Fig. 1). Some of the results provide unexpected insights into the ways in which physicians from different backgrounds approach ICU care. Many tests were used at varying extents by all 4 physician specialties to assess the need for volume expansion, with the 3 most frequently used indicators being blood pressure, urine output, and central venous pressure. Our observations reflected published findings of a Canadian survey (McIntyre et al. 2007) which reported that urine output and blood pressure were also the 2 most commonly cited resuscitation end-points for early septic shock. To our surprise, less invasive parameters, such as pulse pressure or systolic pressure variation and plethysmographic waveform variations, were not frequently used by physicians of any specialty. Of those who chose pulse pressure or systolic pressure variation, anesthesiologists (40 %), critical care medicine specialists (30 %), and pulmonologists (26 %) were more than 2-fold as likely to use these indicators as surgeons (14 %).

**Fig. 2:**
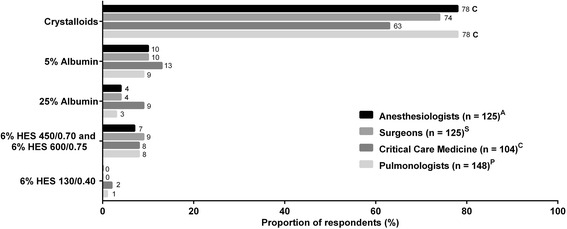
Fluid choices for patient who needs volume expansion but is not bleeding and not septic (patient type 1). First choice of intravenous fluids in response to the question, “Which of the following is your first choice for a patient who needs volume expansion but is not bleeding and not septic?” *Superscripts A*, *S*, *C*, and *P* denote differences between specialties that are statistically significant at *P* < 0.05. HES, hydroxyethyl starch

**Fig. 3:**
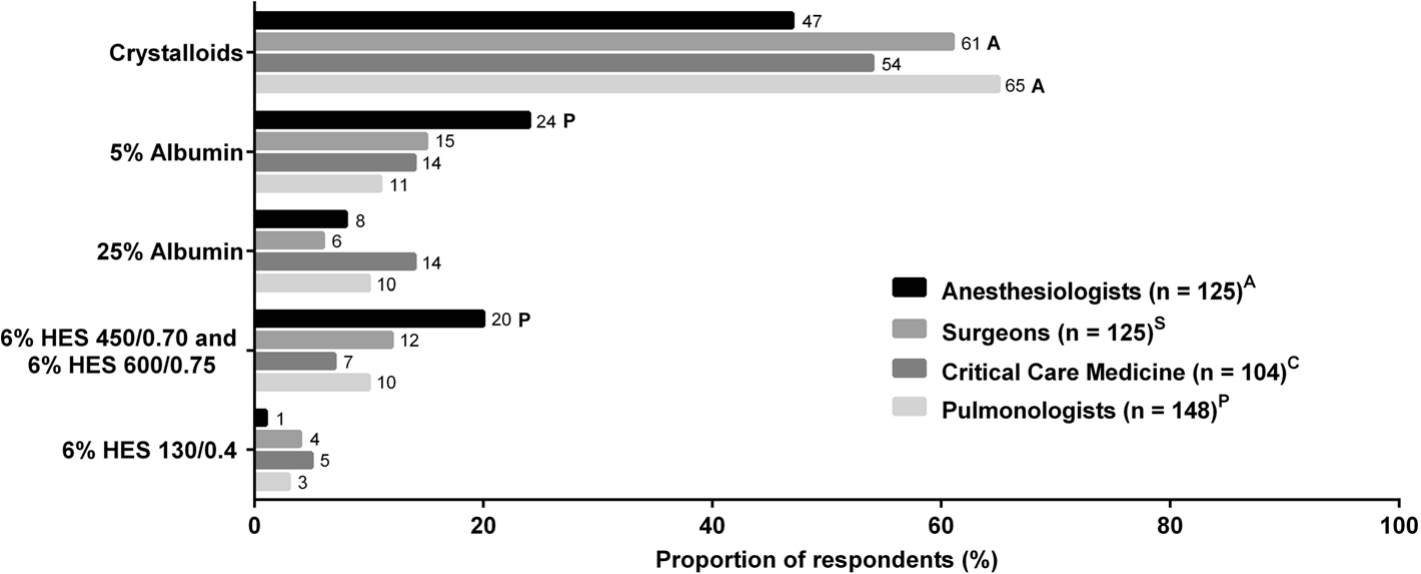
Fluid choices for patient who needs volume expansion in the presence of blood loss when blood transfusion is not indicated (adequate Hb) and patient is not septic (patient type 2). First choice of intravenous fluids in response to the question, “Which of the following is your first choice for a patient who needs volume expansion in the presence of blood loss when blood transfusion is not indicated (adequate Hb) and patient is not septic?” *Superscripts A*, *S*, *C*, and *P* denote differences between specialties that are statistically significant at *P* < 0.05. HES, hydroxyethyl starch

**Fig. 4:**
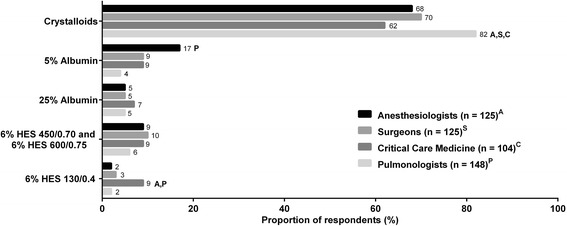
Fluid choices for patient who needs volume expansion for resuscitation in sepsis (patient type 3). First choice of intravenous fluids in response to the question, “Which of the following is your first choice for a patient who needs volume expansion for resuscitation in sepsis?” *Superscripts A*, *S*, *C*, and *P* denote differences between specialties that are statistically significant at *P* < 0.05. HES, hydroxyethyl starch

Overall, crystalloid fluid was the primary choice for fluid resuscitation (Figs. 2, 3, and 4). The second most commonly chosen fluid was 5 % albumin. Only a small fraction of physicians chose 25 % albumin as their first choice, and, therefore, we have elected not to present data on utilization frequency of 25 % albumin in Figs. 2, 3, and 4. Among the 4 clinical specialties of treating physicians, there were subtle, but statistically significant, differences in how fluids were being utilized for all 3 patient types. Data on the frequencies of utilization of various fluids are shown in Additional file 2: Figure S1, Additional file 3: Figure S2, and Additional file 4: Figure S3 for the 3 patient types.

For patient type 1 who needs volume expansion in the *absence of blood loss and sepsis* (Fig. 2), most physicians (63–78 %) chose crystalloids as their first choice of intravenous (IV) fluids regardless of clinical specialty, followed by 5 % albumin (9–13 %). About a quarter of all physicians reported “often” using 5 % albumin and up to 49 % “sometimes” use 5 % albumin (Additional file 2: Figure S1B). HES 450/600 were chosen by 7–9 % of physicians, while only <2 % of physicians chose HES 130. Critical care medicine specialists seemed to prefer less crystalloids and slightly more albumin for this patient type than physicians from other specialties. In contrast to critical care medicine specialists, anesthesiologists most frequently reported “often” choosing crystalloids (Additional file 2: Figure S1A), “sometimes” choosing 5 % albumin (Additional file 2: Figure S1B) and HES 450/600 (Additional file 2: Figure S1C), but “rarely” or “never” HES 130 (Additional file 2: Figure S1D). Interestingly, the highest proportions of those who “rarely” or “never” choose HES 450/600 or HES 130 were pulmonologists.

For patient type 2 who needs volume expansion in *the presence of blood loss but is not septic* (Fig. 3), crystalloid was the first choice of IV fluids (47–65 %), followed by 5 % albumin (11–24 %) and HES 450/600 (7–20 %). Less than 5 % of physicians preferred HES 130. Although anesthesiologists made up the smallest proportion of those who chose crystalloids (47 %) as first choice of fluid in this patient type, they were more likely to choose 5 % albumin (24 %) than pulmonologists (11 %), and they made up the highest proportion (41 %) of those who “often” chose 5 % albumin compared to any other specialties (22–25 %) (Additional file 3: Figure S2B). Surgeons (61 %) and pulmonologists (65 %) were more likely to prefer crystalloids than anesthesiologists (47 %). Moreover, pulmonologists made up the highest proportion of those who reported “always” choosing crystalloid (32 %, Additional file 3: Figure S2A) but “never” HES 450/600 (43 %, Additional file 3: Figure S2C) compared to physicians of any other specialties.

For patient type 3 who needs volume expansion *for resuscitation in sepsis* (Fig. 4), crystalloid was, again, the first-choice of fluids (62–82 %), followed by 5 % albumin (4–17 %). Pulmonologists (82 %) made up the highest proportion of physicians who chose crystalloids as the first choice of fluid. Similar to the patterns observed for patient type 2 (Fig. 3), anesthesiologists (17 %) were more likely to choose 5 % albumin as their first choice than pulmonologists (4 %), and they also made up the highest proportion of those who “often” and “sometimes” choose 5 % albumin (Additional file 4: Figure S3B). Surprisingly, up to 10 % of physicians across all specialties chose HES as first-choice fluids for this patient type despite the 2013 Food and Drug Administration boxed warning against HES use for sepsis due to increased mortality and severe renal injury (U.S. Food and Drug Administration 2013). Furthermore, we did not expect to see more physicians prefer HES 450/600 (6–10 %) over 25 % albumin (5–7 %), as the use of HES for patients with sepsis goes against the Surviving Sepsis Campaign (SSC) recommendations (Dellinger et al. 2013). Among those who chose HES 130 as their first choice, critical care medicine specialists made up the highest proportion (9 %) compared to the 2–3 % of physicians from other specialties.

**Fig. 5:**
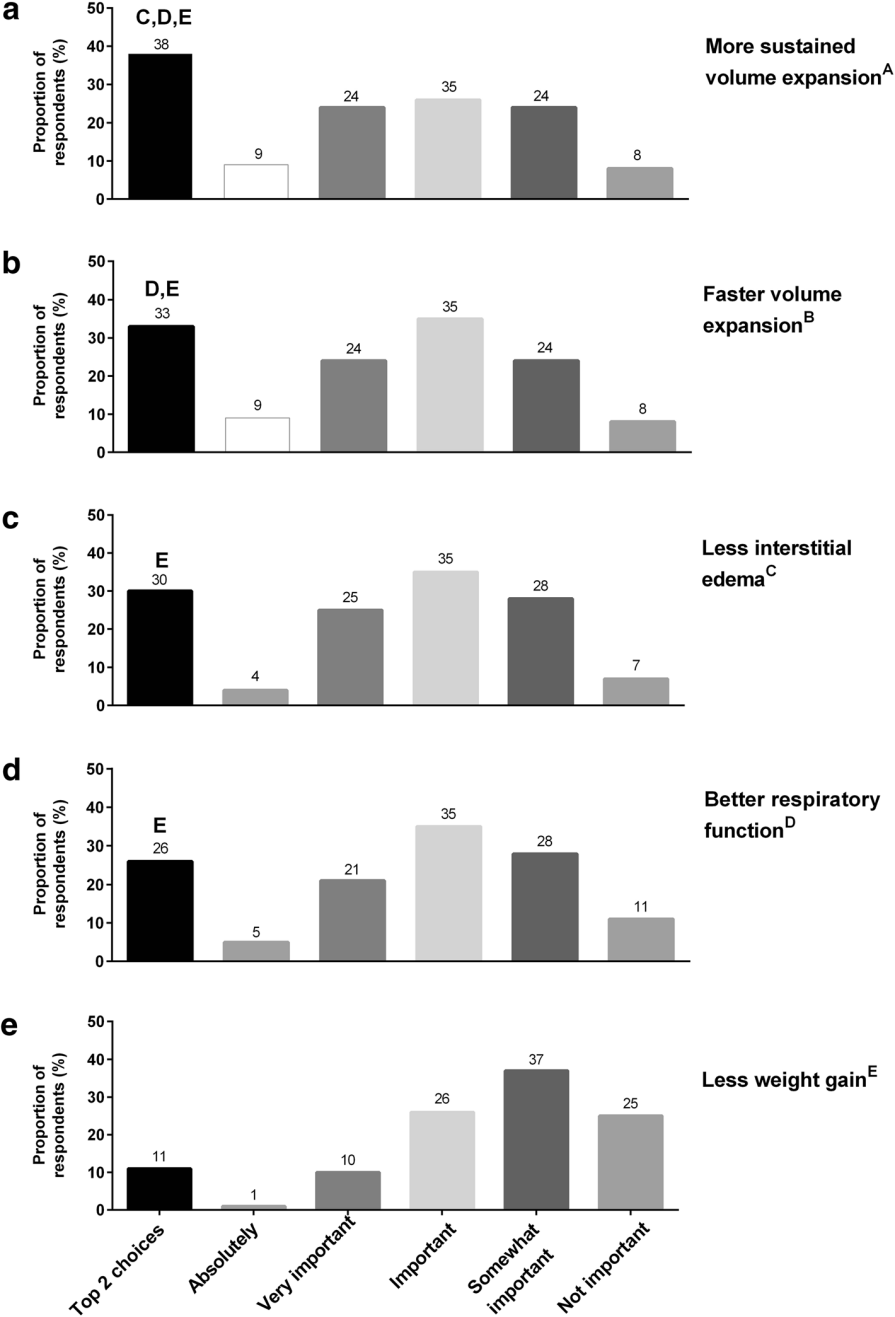
Rankings of the importance of certain colloid traits when colloids are used for volume expansion. Colloid traits assessed are as follows: (**a**) more sustained volume expansion; (**b**) faster volume expansion; (**c**) less interstitial edema; (**d**) better respiratory function; (**e**) less weight gain

When colloids were chosen as the preferred fluid for resuscitation, the 3 reasons most frequently cited for these decisions were the following: (1) more sustained volume expansion (Fig. 5a); (2) faster volume expansion (Fig. 5b); and (3) less interstitial edema (Fig. 5c). “Better respiratory function” appeared to be of less importance (Fig. 5d), and “less weight gain” (Fig. 5e) was the least important factor.

**Fig. 6:**
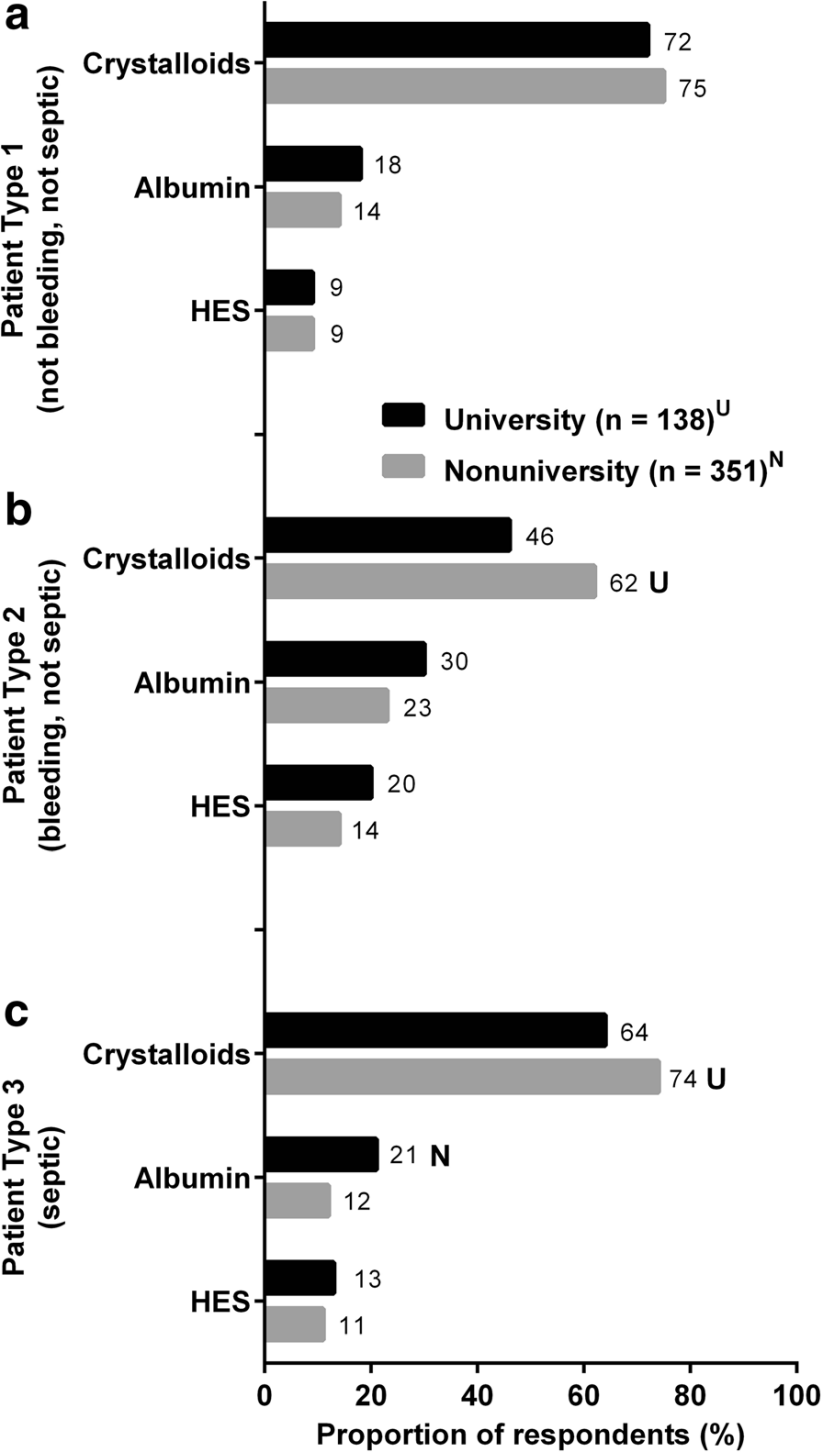
First choice of IV fluids for (**a**) patient type 1, (**b**) patient type 2, and (**c**) patient type 3 as reported by practice settings. “Albumin” group includes 5 % albumin and 25 % albumin. “HES” group includes HES 6 % 450/070, HES 6 % 600/0.75, and HES 6 % 130/0.4. This dataset does not include physicians from Veterans Affairs hospitals due to small sample (*n* = 13). *Superscripts U* and *N* denote differences between practice settings that are statistically significant at *P* < 0.05. HES, hydroxyethyl starch

Another factor affecting the decision-making process was the practice setting from which the physicians came (Fig. 6). Subanalysis results suggest that practice patterns varied depending on whether a physician worked in university vs nonuniversity hospitals. Within the dataset for each patient type, *t* tests were used to assess for the significance of the differences between proportion of physicians from university and nonuniversity hospitals. To simplify the data, we combined 5 % and 25 % albumin into a single category named “albumin.” The same was done for the 3 HES subtypes. Overall, most physicians chose crystalloids as the first choice of fluid regardless of practice setting. However, more physicians from nonuniversity hospitals compared to university hospitals chose crystalloid as their first choice for volume expansion in patient types 2 (bleeding but not septic, Fig. 6b) and 3 (septic, Fig. 6c) but not for patient type 1 (Fig. 6a). For patient type 3, physicians from university hospitals (21 %) preferred albumin more frequently relative to those from nonuniversity hospitals (12 %) (Fig. 6c). This is intriguing, as we wonder how ICU protocols at university hospitals may have played a role in distinguishing the behavior of this group toward treating patients with sepsis.

When we further analyzed data of the physicians who indicated crystalloids as the first-choice fluid based on their clinical specialties (Table 2), we found intriguing differences between how university and nonuniversity hospitals would handle the 3 different types of patients. When the data for all clinical specialties were analyzed together, a higher proportion of physicians from nonuniversity hospitals (61.5 %) chose crystalloids for patient type 2 than those from university hospitals (45.7 %); while no difference in practice setting was found for patient types 1 and 3. When the data were analyzed based on the 4 individual clinical specialties, statistically significant differences were detected for only anesthesiologists and surgeons; while no differences were observed among critical care medicine specialists and pulmonologists based on their practice settings. More anesthesiologists from nonuniversity hospitals than university hospitals preferred crystalloids as their first choice of fluid for patient type 2 (52.1 % vs 32.3 %, respectively) and for patient type 3 (73.4 % vs 51.6 %) but not for patient type 1. Surgeons from university hospitals (45.7 %) chose crystalloid as their first choice less frequently than those from nonuniversity hospitals (66.7 %) for patient type 2.

**Table 2:** Percent of physicians indicating crystalloids as first choice for volume expansion based on patient types

		Practice settings	
		University hospital	Nonuniversity hospital
		(*n* = 138)	(*n* = 351)
All clinical specialties	*n*	138	364
	Patient type 1	71.7 %	74.7 %
	Patient type 2	45.7 %	61.5 %*
	Patient type 3	64.5 %	73.6 %
Anesthesiologists only	*n*	31	94
	Patient type 1	74.2 %	79.8 %
	Patient type 2	32.3 %	52.1 %*
	Patient type 3	51.6 %	73.4 %*
Surgeons only	*n*	35	90
	Patient type 1	65.7 %	76.7 %
	Patient type 2	45.7 %	66.7 %*
	Patient type 3	60.0 %	73.3 %
Critical care medicine only	*n*	33	71
	Patient type 1	60.6 %	64.8 %
	Patient type 2	42.4 %	59.2 %
	Patient type 3	54.5 %	64.8 %
Pulmonologists only	*n*	39	109
	Patient type 1	84.6 %	75.2 %
	Patient type 2	59.0 %	67.0 %
	Patient type 3	87.2 %	79.8 %

Patient type 1: patient who needs volume expansion but is not bleeding and not septic. Patient type 2: patient who needs volume expansion in the presence of blood loss when blood transfusion is not indicated (adequate Hb) and patient is not septic. Patient type 3: patient who needs volume expansion for resuscitation in sepsis

*Statistically significant differences of *P* < 0.05 between practice settings within physician specialty

Interesting observations were also made regarding the preferences for bolus volume. In general, all physicians reported typical volume of colloid bolus (464.4 mL) as smaller than their typical volume of crystalloid bolus (797.4 mL). The estimated ratio of 1:1.7 for albumin to crystalloid volume in our study resembled previously published estimates from the SAFE study (1:1.4) (Finfer et al. 2004) and a systematic review/meta-regression study across 24 reports (1:1.5) (Orbegozo Cortes et al. 2015). When bolus volume was evaluated as a function of clinical practice settings (Table 3), results from a one-sample *t* test indicated that physicians in nonuniversity hospitals appeared to prefer a slightly larger volume of crystalloid bolus (821.7 mL) than do physicians in university hospitals (716.5 mL), although this difference in volume was small and may not have clinical significance in most situations. Data from VA physicians were not included in this particular analysis due to small sample size (*n* = 13). To the best of our knowledge, these results are novel and have not been reported previously.

**Table 3:** Bolus volumes (mL) of fluid resuscitation with colloids vs crystalloids, by practice settings

		University hospitals	Nonuniversity hospitals^a^
		(*n* = 138)	(*n* = 351)
Colloids (mL)	Mean (SD)	411.8 (362.3)	485.5 (504.7)
Crystalloids (mL)	Mean (SD)	716.5 (539.0)	821.7 (612.8)*

*SD* standard deviation

*Statistically significant differences of *P* < 0.05 between practice settings as analyzed by independent-sample *t* test

^a^Excludes physicians in Veterans Affairs hospital setting (*n* = 13)

## Discussion

This survey of 502 physicians from 4 clinical specialties of various ICU practice settings revealed that practice patterns in fluid resuscitation vary broadly with respect to preferences of volume status parameters and preferences for fluid types that would be used to treat patients who need volume expansion but are not bleeding and not septic (patient type 1), patients who need volume expansion due to bleeding but are not septic (patient type 2), and those who need resuscitation due to sepsis (patient type 3). For volume status parameters, the 3 most frequently preferred indicators were blood pressure, urine output, and central venous pressure; whereas, cardiac output, pulse pressure variation, and stroke volume may be considered to be better predictors of hemodynamic response to fluid loading (McDermid et al. 2014). For volume expansion, the most preferred fluid type was crystalloid, followed by 5 % albumin, regardless of the patient type considered. Surprisingly, up to 10 % of physicians still chose HES as a first choice of fluid for resuscitation in sepsis. The clinical specialties and practice settings of the treating physicians also influenced fluid choices. Among the different physician specialties, anesthesiologists appeared to prefer more colloids, particularly 5 % albumin, for patient types 2 and 3 than the other physician subgroups (Figs. 3 and 4). The data suggests that a greater proportion of university physicians than nonuniversity physicians preferred colloid as a first choice of fluids (Fig. 6).

Our finding that crystalloid was the first choice of fluid for most physicians in the USA aligned with published data from an international, cross-sectional survey (391 ICUs) in 2007 (Finfer et al. 2010), which found that the USA and New Zealand were more likely to use crystalloids than colloids; whereas, countries like Australia, China, Great Britain, Switzerland, and Sweden used more colloids than crystalloids. Furthermore, the strongest determinant of fluid choice was location of country, not measures of illness severity in their patients. Although we did not measure variations among geographic regions of the USA, we did observe differences in fluid choice based on clinical practice settings. Physicians from the nonuniversity hospitals preferred crystalloids more than those from university hospitals for all 3 patient types (Fig. 6).

Specifically for patients with sepsis (patient type 3), physicians from university hospitals were the more likely to prefer albumin compared to physicians from nonuniversity hospitals. Of interest for septic patients, HES was chosen by 11 %–13 % of physicians (Fig. 6c); this was unexpected because the use of HES in septic patients does not align with SSC recommendations (Dellinger et al. 2013). Based on results from the VISEP (Brunkhorst et al. 2008), 6S (Perner et al. 2012), and CHEST (Myburgh et al. 2012) trials, the SSC recommended against the use of HES. Instead, fluid resuscitation in sepsis as defined by the SSC includes initial fluid challenge with crystalloids as the first choice of fluid (grade 1B), followed by albumin as the second choice if patients are unresponsive to large amounts of crystalloids. When compared to other fluid types, HES solutions, regardless of molecular weight, may be associated with increased mortality and acute kidney injury in patients with sepsis as well as in the general population (Myburgh et al. 2012; Brunkhorst et al. 2008; Guidet et al. 2012; Schortgen et al. 2001; Zarychanski et al. 2013).

More physicians across all specialties chose colloid for patients with blood loss (patient type 2) than for the other 2 patient examples. Anesthesiologists preferred more colloid, both 5 % albumin and HES, than physicians from other specialties. This may be because anesthesiologists, relative to other specialists, are more familiar with managing blood loss, when the faster speed of shock reversal with colloid with less fluid volume required may be particularly advantageous (Rehm et al. 2000; Roger et al. 2014).

When asked about the importance of certain colloid traits when colloids are used for volume expansion, 60 % of physicians thought that the more sustained volume expansion, faster volume expansion, and less interstitial edema with colloids were either important or very important (Fig. 5), suggesting that physicians are generally aware of the potential physiological advantages of colloids. Only 8 % of respondents thought that these properties were not important. With respect to the use of albumin solutions as a specific type of colloid fluid, our data aligns with previously published data from another cross-sectional survey of 61 ICUs in Australia and New Zealand between 2007 and 2013 (Hammond et al. 2015), which found that hypo-oncotic albumin (31.6 %) was used more frequently than hyper-oncotic albumin (11.6 %). When comparing the fluid preference for resuscitation in sepsis, our data showed that physicians in the USA preferred more crystalloids over colloids (Fig. 4).

Recent publications are calling for IV fluids to be considered as drugs and for physicians to approach fluid infusions with the same considerations one would give for the pharmacokinetic and pharmacodynamic characteristics of any other treatment (Rewa and Bagshaw 2015; McDermid et al. 2014; Santi et al. 2015; Severs et al. 2015). Furthermore, Bellomo and colleagues (Bellomo et al. 2006) reported that fluid volume may be an independent predictor of changes in pH, chloride, ionized calcium, bicarbonate, base excess, and effective strong ion difference within the first 24 h of resuscitation.

Our study revealed that physicians reported using a wide range of fluid volumes for fluid boluses regardless of clinical practice settings. Published evidence indicates that initial high-volume fluid resuscitation is associated with increased overall mortality in patients with trauma-related bleeding (Wang et al. 2014) and that hyper-oncotic albumin fluids are suitable for small-volume resuscitation with no evidence of deleterious effects (Jacob et al. 2008). Evidence from well-designed network meta-analyses and clinical trials suggests that crystalloid fluids, particularly balanced solutions, seem to be the most advisable first choice, and albumin is equivalent or superior to other available fluids in severe sepsis and septic shock (Jacob et al. 2008; Rochwerg et al. 2015; Rochwerg et al. 2014; Delaney et al. 2011; SAFE Study Investigators et al. 2011). Altogether, the data from this survey suggest that the participating physicians may prefer albumin when a smaller bolus volume is desired to achieve a more rapid and sustained volume expansion. Thus in the context of sepsis, we believe it makes sense that using small-volume fluid resuscitation, particularly with colloids (e.g., albumin) early in the course of the disease before significant EGL damage ensues, may be better at limiting fluid loss into the interstitium, leading to better intravascular volume expansion. Unfortunately, our data do not explain how physicians would approach the decision on bolus size based on the disease context.

The strength of this study is that it provides an up-to-date picture of how fluid management is practiced in the USA. Although a few international surveys on fluid resuscitation (McIntyre et al. 2007; Finfer et al. 2010; Schortgen et al. 2004) have been conducted in the past, major changes in fluid management have occurred in recent years (Hammond et al. 2015), and past data may no longer be relevant. For instance, we note that an older survey conducted by the CRYCO Study Group in 2002 showed that colloids, not crystalloids, were widely chosen as the first choice of fluid in Europe (Schortgen et al. 2004), and starches were the most frequently used colloids during that time. A limitation of this study is that it describes current practices in only the USA. We acknowledge that our study results cannot be generalized to other countries in the rest of the world. As our study is based on a survey of physician preferences, we did not report on clinical outcomes. We also recognize that the decision-making processes of physicians are complex and may vary from the situations we put forth in the survey. While our study design cannot provide details for all of the reasons underlying treatment decisions, the results allow physicians to compare their own practice patterns to other colleagues, which could be helpful given the lack of expert consensus on fluid resuscitation in critically ill patients. It is also important to provide basic background information for the design of future trials that address complicated issues, such as fluid resuscitation strategies. Although appropriate timing of fluid administration is an important topic, our survey was not designed to delineate this matter; thus, we cannot comment on how fluid resuscitation is influenced by the temporal cadence of ICUs in the USA. Based on the published literature, one could hypothesize that early fluid therapy with colloids followed by a restricted volume strategy may be associated with better outcomes (van Haren and Zacharowski 2014), especially in the context of sepsis (Lee et al. 2014).

## Conclusions

This survey of physicians from different specialties caring for patients in ICUs of the USA highlights several important issues in fluid resuscitation and fluid management, which should be based on the underlying pathophysiology of the disease context of the patient. Fluid resuscitation is one component of a complex resuscitation process with changing requirements over the duration of acute illness. However, estimating the extent of hypovolemia or evaluating the response to a fluid challenge is difficult, as there is no single clinical or biochemical parameter that reflects the complexity of the circulation, particularly under rapidly changing pathological conditions. With the lack of consensus, the physicians in our study approached fluid resuscitation in a wide variety of ways, depending on their clinical specialties and practice settings. It is important for physicians to keep in mind the variability in fluid requirements of each patient necessitates a targeted and integrated assessment of volume status and on-going fluid needs (Rewa and Bagshaw 2015). Crystalloid solution is the primary fluid of choice for nonseptic and septic patients. If physicians wish to use colloids in septic patients, then according to the FDA and SSC recommendations albumin would be the logical second choice, not HES, due to recent concerns about HES safety (U.S. Food and Drug Administration 2013; Dellinger et al. 2013; Perner et al. 2012). In our survey, colloids were used more frequently in the bleeding patient type, because physicians felt that colloids provide more rapid and sustained volume expansion (Perner et al. 2012). We encourage physicians to prescribe fluids as one would any other drug, carefully considering the fluid amount, the fluid type, and the disease context specific to the patient being treated.

## Additional files

Additional file 1: ICU Fluid Utilization Survey. (DOCX 308 kb) Additional file 2: Figure S1. Fluid choices for patient who needs volume expansion but is not bleeding and not septic (patient type 1). As follow-up questions (for “Which of the following is your first choice for a patient who needs volume expansion but is not bleeding and not septic?”), the utilization frequency of (A) crystalloid, (B) 5 % albumin, (C) 6 % HES 450/0.70 AND 6 % HES 600/0.75, and (D) 6 % HES 130/0.40 was assessed by asking the question, “How often do you use each of the following in a patient when volume expansion is indicated in the absence of blood loss and sepsis?” *N* values for panels A–D are as follows: anesthesiologists (*n* = 125), surgeons (*n* = 121), critical care medicine (*n* = 98), pulmonologists (*n* = 146). Superscripts A, S, C, and P denote differences between specialties that are statistically significant at *P* < 0.05. HES, hydroxyethyl starch. (JPG 1674 kb) Additional file 3: Figure S2. Fluid choices for patient who needs volume expansion in the presence of blood loss when blood transfusion is not indicated (adequate Hb) and patient is not septic (patient type 2). As follow-up questions (for “Which of the following is your first choice for a patient who needs volume expansion in the presence of blood loss when blood transfusion is not indicated (adequate Hb) and patient is not septic?”), the utilization frequency of (A) crystalloid, (B) 5 % albumin, (C) 6 % HES 450/0.70 AND 6 % HES 600/0.75, and (D) 6 % HES 130/0.40 was assessed by asking the question, “How often do you use each of the following in a patient for volume expansion in the presence of blood loss when blood transfusion is not indicated (adequate Hb) and patient is not septic?” *N* values for panels A–D are as follows: anesthesiologists (*n* = 125), surgeons (*n* = 121), critical care medicine (*n* = 98), pulmonologists (*n* = 146). Superscripts A, S, C, and P denote differences between specialties that are statistically significant at *P* < 0.05. HES, hydroxyethyl starch. (JPG 1692 kb) Additional file 4: Figure S3. Fluid choices for patient who needs volume expansion for resuscitation in sepsis (patient type 3). As follow-up questions (for “Which of the following is your first choice for a patient who needs volume expansion for resuscitation in sepsis?” As follow-up questions, the utilization frequency of (A) crystalloid, (B) 5 % albumin, (C) 6 % HES 450/0.70 AND 6 % HES 600/0.75, and (D) 6 % HES 130/0.40 was assessed by asking the question, “How often do you use each of the following or a patient who needs volume expansion for resuscitation in sepsis?” *N* values for panels A–D are as follows: anesthesiologists (*n* = 125), surgeons (*n* = 120), critical care medicine (*n* = 98), pulmonologists (*n* = 146). Superscripts A, S, C, and P denote differences between specialties that are statistically significant at *P* < 0.05. HES, hydroxyethyl starch. (JPG 1701 kb)

## Abbreviations

6S: Scandinavian Start for Severe Sepsis/Septic Shock Study
ALBIOS: Albumin Italian Outcome Sepsis Study
CHEST: Crystalloid Versus Hydroxyethyl Starch Trial
CRISTAL: Colloids Versus Crystalloids for the Resuscitation of the Critically Ill Trial
EGL: endothelial glycocalyx layer
FIRST: Fluids in Resuscitation of Severe Trauma Trial
Hb: hemoglobin
HES: hydroxyethyl starches
ICU: intensive care unit
IV: intravenous
SAFE: Saline and Albumin Fluid Evaluation Trial
SSC: Surviving Sepsis Campaign
VISEP: Efficacy of Volume Substitution and Insulin Therapy in Severe Sepsis Study
