# Supplementary material for: Fluid resuscitation practice patterns in intensive care units of the USA: a cross-sectional survey of critical care physicians
Source: Perioper Med (Lond). 2016 Jun 16;5:15. doi: 10.1186/s13741-016-0035-2 (PMC4910257; doi:10.1186/s13741-016-0035-2)
Supplement: Additional file 1: — ICU Fluid Utilization Survey. (DOCX 308 kb) [file 13741_2016_35_MOESM1_ESM.docx]

**ICU Albumin Utilization Survey**

Final Version, 2015

**Part 1: Demographics**

1. Which of the following is your primary specialty?
   1. Anesthesiology
   2. Surgery
   3. Critical Care Medicine
   4. Pediatrics
   5. Pulmonology
   6. Other (Please specify _______________________________________________)
2. In which state or region is your primary practice located?

[Drop-down list of states]

[EXCLUDE / TERM PHYSICIANS IN Minnesota (MN), Vermont (VT), West Virginia (WVA), District of Columbia (DC) AND Massachusetts (MA)]

1. In which of the following intensive care units do you work? (If you work in more than one, please select the unit type that best describes the one in which you most frequently work.)
   1. Cardiac ICU
   2. Neuro ICU
   3. Surgical ICU
   4. Pediatric ICU
   5. Medical ICU
   6. ICU accepting a variety of patients
   7. I do not work in an ICU. [EXIT]
2. What year did you finish your residency or fellowship training?

[Drop-down list of years] [RANGE 1951-2015]

[TERM IF 2013/2014/2015]

QUALIFYING CRITERIA: RESPONDENTS MUST MEET THE FOLLOWING SCREENING CRITERIA TO CONTINUE IN THE SURVEY:

1. Specialty must be in Anesthesiology, Surgery, Critical Care Medicine, or Pulmonology (Q1=a, b, c, or e)
2. Practice must not be in one of the excluded states/areas listed in Q2
3. Must work in Surgical ICU or Medical ICU (Q3=c or e or f)

Surgical ICU quota: if S1= a, b, c, or e AND if S3=c

OR if S1=a or b AND if S3=f

Medical ICU quota: if S1= a, b, c, or e and if S3=e

OR if S1=c or e and if S3=f

D. Must have been in practice at least two years since residency (based on Q4)

| **Sample goal** |  | |
| --- | --- | --- |
|  | **Medical ICU** | **Surgical ICU** |
| **Physician specialty** | (n) | (n) |
| Pulmonology | 175 | -- |
| Critical Care Medicine | 75 | -- |
| Anesthesiology | -- | 125 |
| Surgeon | -- | 125 |
| **ICU totals** | **250** | **250** |
| **Sample totals** | **n=500** | |

1. Which of the following best describes your primary practice setting?
   1. University Hospital
   2. Non-teaching Hospital
   3. Private Practice
   4. Community Hospital
   5. VA Hospital

[DISPLAY Q6 AND Q7 ON SAME SCREEN]

1. Approximately how many beds in total does your primary hospital have?

[RANGE 0001 – 3000]

|_|_|_|_| beds in total

1. How many **intensive care unit** beds does your primary hospital have?

[RANGE 0001 – 2000]

|_|_|_|_| **intensive care unit** beds

**Part 2: Fluid and hemodynamic management**

1. This brief survey focuses on fluid and hemodynamic management in the ICU. Please answer all questions as accurately as you can.

Using the scale below, please indicate how frequently you use each of the following fluids for **hourly maintenance**, if at all.

|  | Always | Often | Sometimes | Rarely | Never |
| --- | --- | --- | --- | --- | --- |
| [RANDOMIZE ORDER] | 1 | 2 | 3 | 4 | 5 |
| NS (Normal saline) |  |  |  |  |  |
| DS (Dextrose saline) |  |  |  |  |  |
| LR (Lactated Ringer's solution) |  |  |  |  |  |
| Normosol/plasmalyte |  |  |  |  |  |

1. Which of the following indicators (diagnostic tools) of volume status and the need for volume expansion do you use? (Please select all that apply.)
   1. Blood pressure
   2. Cardiac output
   3. Central venous pressure
   4. Central venous saturation (SvO2)
   5. Global end diastolic volume
   6. Mixed venous saturation (ScvO2)
   7. Plethysmographic Waveform Variation
   8. Pulmonary capillary wedge pressure
   9. Pulse Pressure Variation or Systolic Pressure Variation
   10. Stroke Volume Variation
   11. Transesophageal echocardiography
   12. Transthoracic echocardiography
   13. Urine output
   14. None. I depend solely on my clinical experience. [SKIP TO Q3]
   15. None of the above [SKIP TO Q3]

[ASK Q2B IF Q2/A through M]

2b. How frequently do you use each of the volume expansion indicators listed below?

|  | Always | Often | Sometimes | Rarely | Never |
| --- | --- | --- | --- | --- | --- |
| [DISPLAY ONLY INDICATORS SELECTED IN Q2.] [DISPLAY IN SAME ORDER AS IN Q2] | 1 | 2 | 3 | 4 | 5 |
| Blood pressure |  |  |  |  |  |
| Cardiac output |  |  |  |  |  |
| Central venous pressure |  |  |  |  |  |
| Central venous saturation (SvO2) |  |  |  |  |  |
| Global end diastolic volume |  |  |  |  |  |
| Mixed venous saturation (ScvO2) |  |  |  |  |  |
| Plethysmographic Waveform Variation |  |  |  |  |  |
| Pulmonary capillary wedge pressure |  |  |  |  |  |
| Pulse Pressure Variation or Systolic Pressure Variation |  |  |  |  |  |
| Stroke Volume Variation |  |  |  |  |  |
| Transesophageal echocardiography |  |  |  |  |  |
| Transthoracic echocardiography |  |  |  |  |  |
| Urine output |  |  |  |  |  |

1. Which of the following is your first choice for a patient who needs volume expansion but is not bleeding and not septic?

[RANDOMIZE ORDER]

- Albumin 5%

Keep/display together

Keep/display together

- Albumin 25%
- HES – Voluven
- HES – hextend/hespan
- Crystalloids
- None; not appropriate for my practice [ANCHOR; SKIP TO Q5]

1. How often do you use each of the following in a patient when volume expansion is indicated in the absence of blood loss and sepsis?

|  | Always | Often | Sometimes | Rarely | Never |
| --- | --- | --- | --- | --- | --- |
|  | 1 | 2 | 3 | 4 | 5 |
| Albumin 5% |  |  |  |  |  |
| Albumin 25% |  |  |  |  |  |
| HES - Voluven |  |  |  |  |  |
| HES – hextend/hespan |  |  |  |  |  |
| Blood derived products other than albumin |  |  |  |  |  |
| Only crystalloids |  |  |  |  |  |

1. Which of the following is your first choice for a patient who needs volume expansion in the presence of blood loss when blood transfusion is not indicated (adequate Hb) and patient is not septic?

[RANDOMIZE ORDER]

- Albumin 5%

Keep/display together

Keep/display together

- Albumin 25%
- HES – Voluven
- HES – hextend/hespan
- Crystalloids
- None; not appropriate for my practice [ANCHOR; SKIP TO Q7]

1. How often do you use each of the following in a patient for volume expansion in the presence of blood loss when blood transfusion is not indicated (adequate Hb) and patient is not septic?

|  | Always | Often | Sometimes | Rarely | Never |
| --- | --- | --- | --- | --- | --- |
|  | 1 | 2 | 3 | 4 | 5 |
| Albumin 5% |  |  |  |  |  |
| Albumin 25% |  |  |  |  |  |
| HES - Voluven |  |  |  |  |  |
| HES – hextend/hespan |  |  |  |  |  |
| Blood derived products other than albumin |  |  |  |  |  |
| Only crystalloids |  |  |  |  |  |

1. Which of the following is your first choice for a patient who needs volume expansion for resuscitation in sepsis?

[RANDOMIZE ORDER]

- Albumin 5%

Keep/display together

Keep/display together

- Albumin 25%
- HES – Voluven
- HES – hextend/hespan
- Crystalloids
- None; not appropriate for my practice [ANCHOR; SKIP TO Q9]

1. How often do you use each of the following for a patient who needs volume expansion for resuscitation in sepsis?

|  | Always | Often | Sometimes | Rarely | Never |
| --- | --- | --- | --- | --- | --- |
|  | 1 | 2 | 3 | 4 | 5 |
| Albumin 5% |  |  |  |  |  |
| Albumin 25% |  |  |  |  |  |
| HES - Voluven |  |  |  |  |  |
| HES – hextend/hespan |  |  |  |  |  |
| Blood derived products other than albumin |  |  |  |  |  |
| Only crystalloids |  |  |  |  |  |

1. Using the scale below, please indicate how important each of the following is in terms of your reasons for using colloids for volume expansion.

|  | Not Important | Somewhat Important | Important | Very Important | Absolutely Essential |
| --- | --- | --- | --- | --- | --- |
| [RANDOMIZE ORDER] | 1 | 2 | 3 | 4 | 5 |
| More sustained volume expansion with colloids |  |  |  |  |  |
| Faster volume expansion with colloids |  |  |  |  |  |
| Less interstitial edema with colloids |  |  |  |  |  |
| Less weight gain with colloids |  |  |  |  |  |
| Better respiratory function with colloids |  |  |  |  |  |

[ASK Q10 IF TWO OR MORE PROPERTIES IN Q9 ARE RATED EQUALLY AND RATED MOST IMPORANT RELATIVE TO OTHER PROPERTIES. ASK Q11 IF ONE PROPERTY IS RANKED HIGHER THAN OTHER PROPERTIES] ERROR MESSAGE: “Please revise your responses on this page and select only one item per rank order.”

1. Please rank order the following in terms of their importance to your reasons for using colloids for volume expansion, with 1 indicating the “Most important” reason.

SHOW ONLY NUMBER OF RANK POSITIONS EQUAL TO NUMBER OF PROPERTIES SHOWN

| [SHOW ONLY PROPERTIES FROM Q9 RATED  Most Important  2  1  EQUALLY AND RATED MOST IMPORTANT  RELATIVE TO OTHER PROPERTIES] | 3 | 4 | 5 |
| --- | --- | --- | --- |
| [DISPLAY IN SAME ORDER AS IN Q9] |  |  |  |
| More sustained volume expansion with colloids |  |  |  |
| Faster volume expansion with colloids |  |  |  |
| Less interstitial edema with colloids |  |  |  |
| Less weight gain with colloids |  |  |  |
| Better respiratory function with colloids |  |  |  |

1. Which of the following do you most often use to bolus for volume expansion?

[ROTATE ORDER]

a. Colloid

b. Crystalloid

12a. When indicated, what volume of colloid bolus do you typically use for volume expansion?

[RANGE 001 – 9999]

|_|_|_|_| ml

12b. When indicated, what volume of crystalloid bolus do you typically give for volume expansion?

[RANGE 001 – 9999]

|_|_|_|_| ml

1. How common is it in your practice to see each of following adverse reactions to albumin?

| [RANDOMIZE ORDER] | Common  (1 in 10) | Rare  (1 in 100) | | Very Rare  (1 in 1000+) | | Have never seen it | |  |
| --- | --- | --- | --- | --- | --- | --- | --- | --- |
| Rash |  | |  | |  | |  | |
| Hypotension |  | |  | |  | |  | |
| Anaphylaxis |  | |  | |  | |  | |

1. Using the scale below, please indicate how important each of the following non-oncotic properties of albumin is to you in treating your patients.

| Not  Important | | | Somewhat Important | | Important | | Very  Important | | Absolutely Essential | | I am not aware of this property | |
| --- | --- | --- | --- | --- | --- | --- | --- | --- | --- | --- | --- | --- |
| [RANDOMIZE ORDER] 1 | | | 2 | | 3 | | 4 | | 5 | | 99 | |
| Transport of metabolites | | |  | |  | |  | |  | |  | |
| Free radical scavenging | | |  | |  | |  | |  | |  | |
| Anti-inflammatory effects | | |  | |  | |  | |  | |  | |
| Maintenance of vascular integrity | | |  | |  | |  | |  | |  | |
| Antithrombotic effects | | |  | |  | |  | |  | |  | |
| Drug binding |  |  | |  | |  | |  | |  | |  |

[ASK Q15 IF TWO OR MORE PROPERTIES IN Q14 ARE RATED EQUALLY AND RATED MOST IMPORANT RELATIVE TO OTHER PROPERTIES. ASK Q16 IF ONE PROPERTY IS RANKED HIGHER THAN OTHER PROPERTIES] DO NOT INCLUDE ANY PROPERTY RANKED 99. ERROR MESSAGE: “Please revise your responses on this page and select only one item per rank order.”

1. Please rank the following non-oncotic properties of albumin in order of their importance to you in treating your patients, with 1 indicating the “Most important” reason.

SHOW ONLY NUMBER OF RANK POSITIONS EQUAL TO NUMBER OF PROPERTIES SHOWN

|  | | | Most  Important | |  |  | |  | | |  |  | |  |
| --- | --- | --- | --- | --- | --- | --- | --- | --- | --- | --- | --- | --- | --- | --- |
| [SHOW ONLY PROPERTIES FROM Q14 RATED EQUALLY AND RATED MOST IMPORTANT RELATIVE TO OTHER PROPERTIES] | | | 1 | | 2 | 3 | | 4 | | | 5 | 6 | |  |
| Transport of metabolites | | |  | |  |  | |  | | |  |  | |  |
| Free radical scavenging | | |  | |  |  | |  | | |  |  | |  |
| Anti-inflammatory effects | | |  | |  |  | |  | | |  |  | |  |
| Maintenance of vascular integrity | | |  | |  |  | |  | | |  |  | |  |
| Antithrombotic effects | | |  | |  |  | |  | | |  |  | |  |
| Drug binding |  |  | |  | | |  | |  |  | | |  | |

[ASK 15B IF Q4_1=5 (SELECTED “NEVER” FOR “ALBUMIN 5%” IN Q4]

15B. You indicated that you never use albumin 5% for a patient who needs volume expansion but is not bleeding and not septic.

Please briefly tell us why you never use albumin 5% for this type of patient.

[Large text box]

[ASK 15D IF Q6_1=5 (SELECTED “NEVER” FOR “ALBUMIN 5%” IN Q6]

15D. You indicated that you never use albumin 5% for a patient who needs volume expansion in the presence of blood loss when blood transfusion is not indicated (adequate Hb) and patient is not septic.

Please briefly tell us why you never use albumin 5% for this type of patient.

[Large text box]

[ASK 15C IF Q8_1=5 (SELECTED “NEVER” FOR “ALBUMIN 5%” IN Q8]

15C. You indicated that you never use albumin 5% for a patient who needs volume expansion for resuscitation in sepsis.

Please briefly tell us why you never use albumin 5% for this type of patient.

[Large text box]

1. Thank you for your interest and time in completing this survey. [END]
