# Supplementary figures and images for: Fluid resuscitation practice patterns in intensive care units of the USA: a cross-sectional survey of critical care physicians
Source: Perioper Med (Lond). 2016 Jun 16;5:15. doi: 10.1186/s13741-016-0035-2 (PMC4910257; doi:10.1186/s13741-016-0035-2)

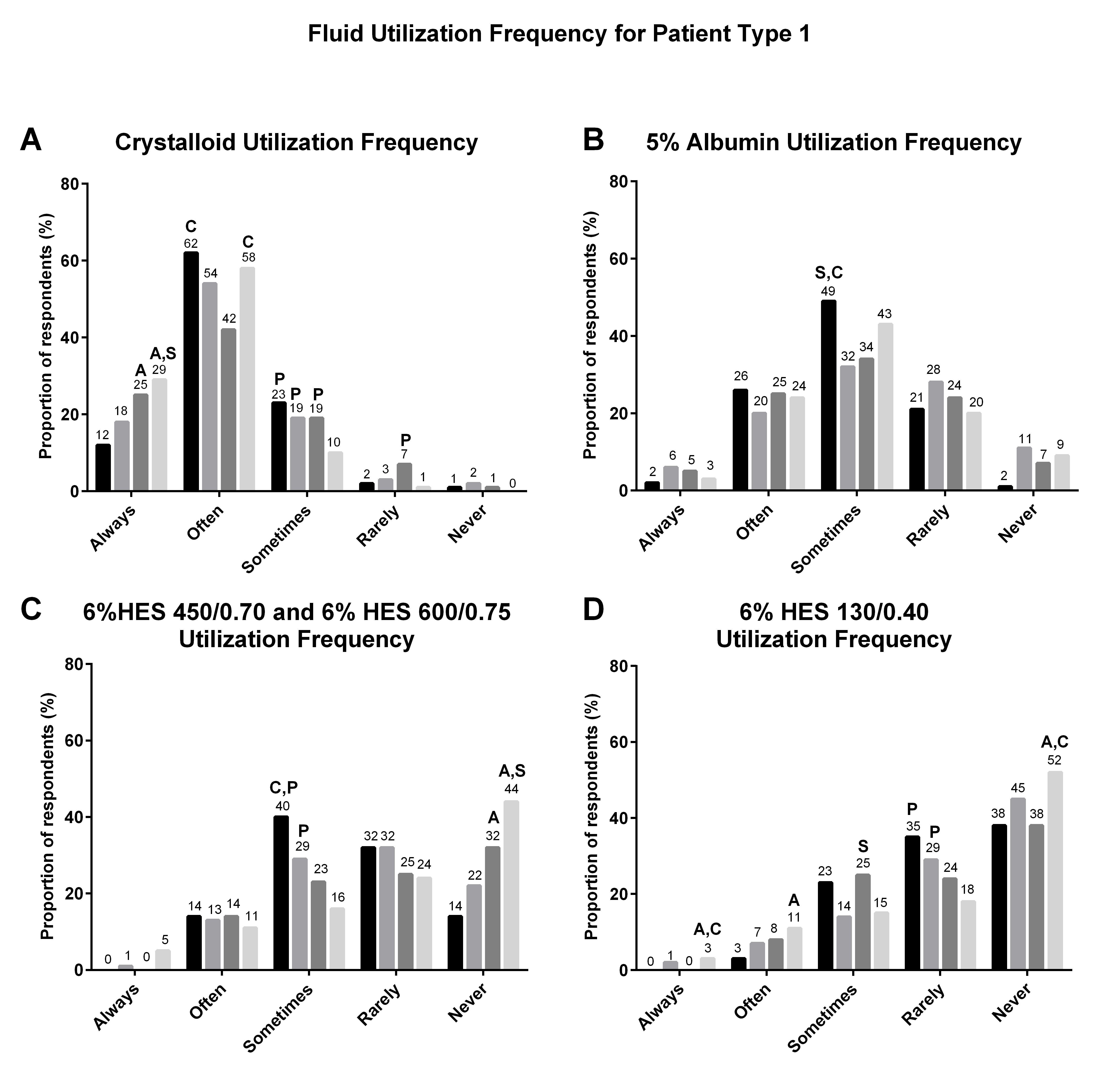

Supplement: Additional file 2: Figure S1. — Fluid choices for patient who needs volume expansion but is not bleeding and not septic (patient type 1). As follow-up questions (for “Which of the following is your first choice for a patient who needs volume expansion but is not bleeding and not septic?”), the utilization frequency of (A) crystalloid, (B) 5 % albumin, (C) 6 % HES 450/0.70 AND 6 % HES 600/0.75, and (D) 6 % HES 130/0.40 was assessed by asking the question, “How often do you use each of the following in a patient when volume expansion is indicated in the absence of blood loss and sepsis?” N values for panels A–D are as follows: anesthesiologists (n = 125), surgeons (n = 121), critical care medicine (n = 98), pulmonologists (n = 146). Superscripts A, S, C, and P denote differences between specialties that are statistically significant at P < 0.05. HES, hydroxyethyl starch. (JPG 1674 kb) [file 13741_2016_35_MOESM2_ESM.jpg]

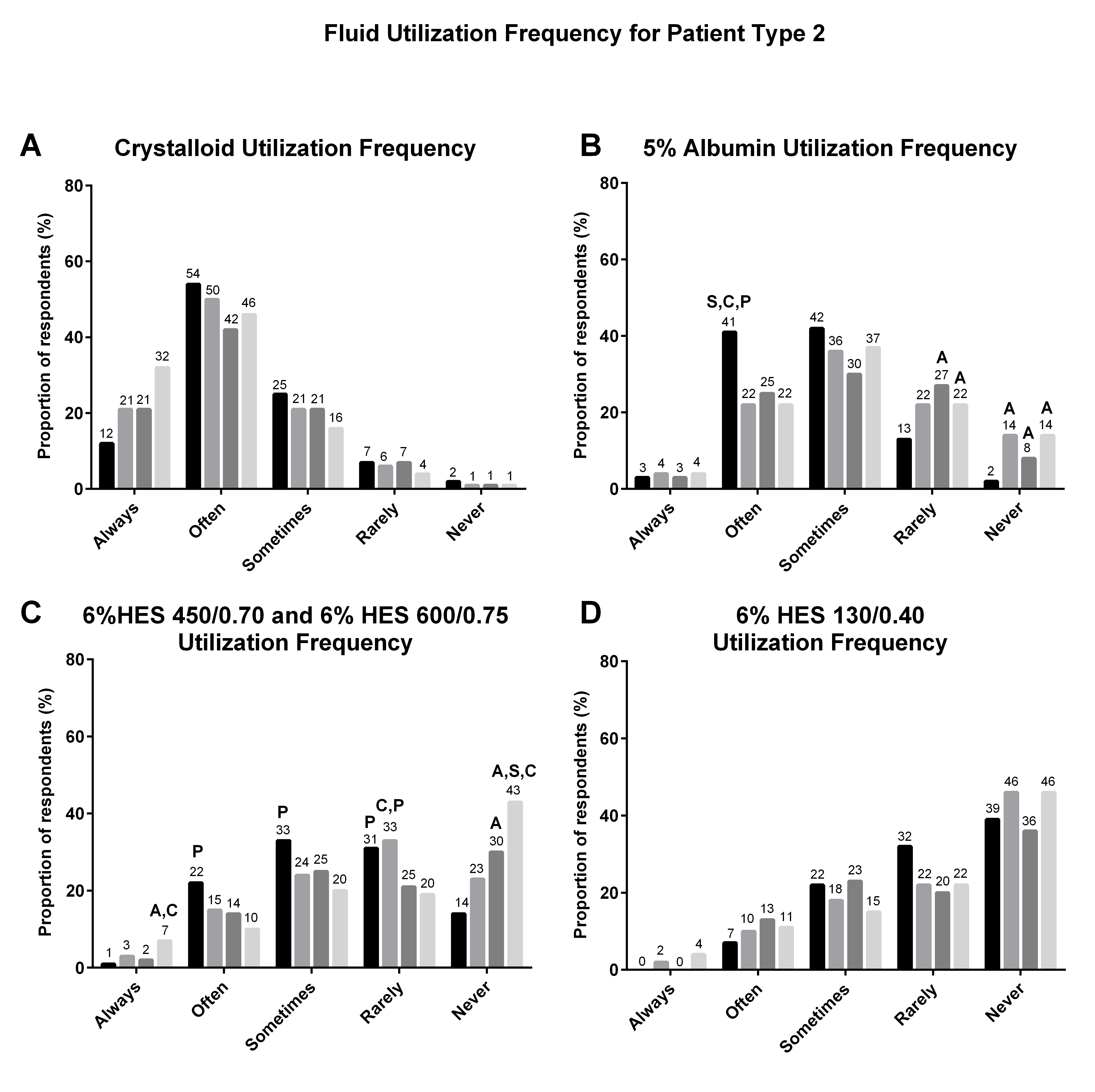

Supplement: Additional file 3: Figure S2. — Fluid choices for patient who needs volume expansion in the presence of blood loss when blood transfusion is not indicated (adequate Hb) and patient is not septic (patient type 2). As follow-up questions (for “Which of the following is your first choice for a patient who needs volume expansion in the presence of blood loss when blood transfusion is not indicated (adequate Hb) and patient is not septic?”), the utilization frequency of (A) crystalloid, (B) 5 % albumin, (C) 6 % HES 450/0.70 AND 6 % HES 600/0.75, and (D) 6 % HES 130/0.40 was assessed by asking the question, “How often do you use each of the following in a patient for volume expansion in the presence of blood loss when blood transfusion is not indicated (adequate Hb) and patient is not septic?” N values for panels A–D are as follows: anesthesiologists (n = 125), surgeons (n = 121), critical care medicine (n = 98), pulmonologists (n = 146). Superscripts A, S, C, and P denote differences between specialties that are statistically significant at P < 0.05. HES, hydroxyethyl starch. (JPG 1692 kb) [file 13741_2016_35_MOESM3_ESM.jpg]

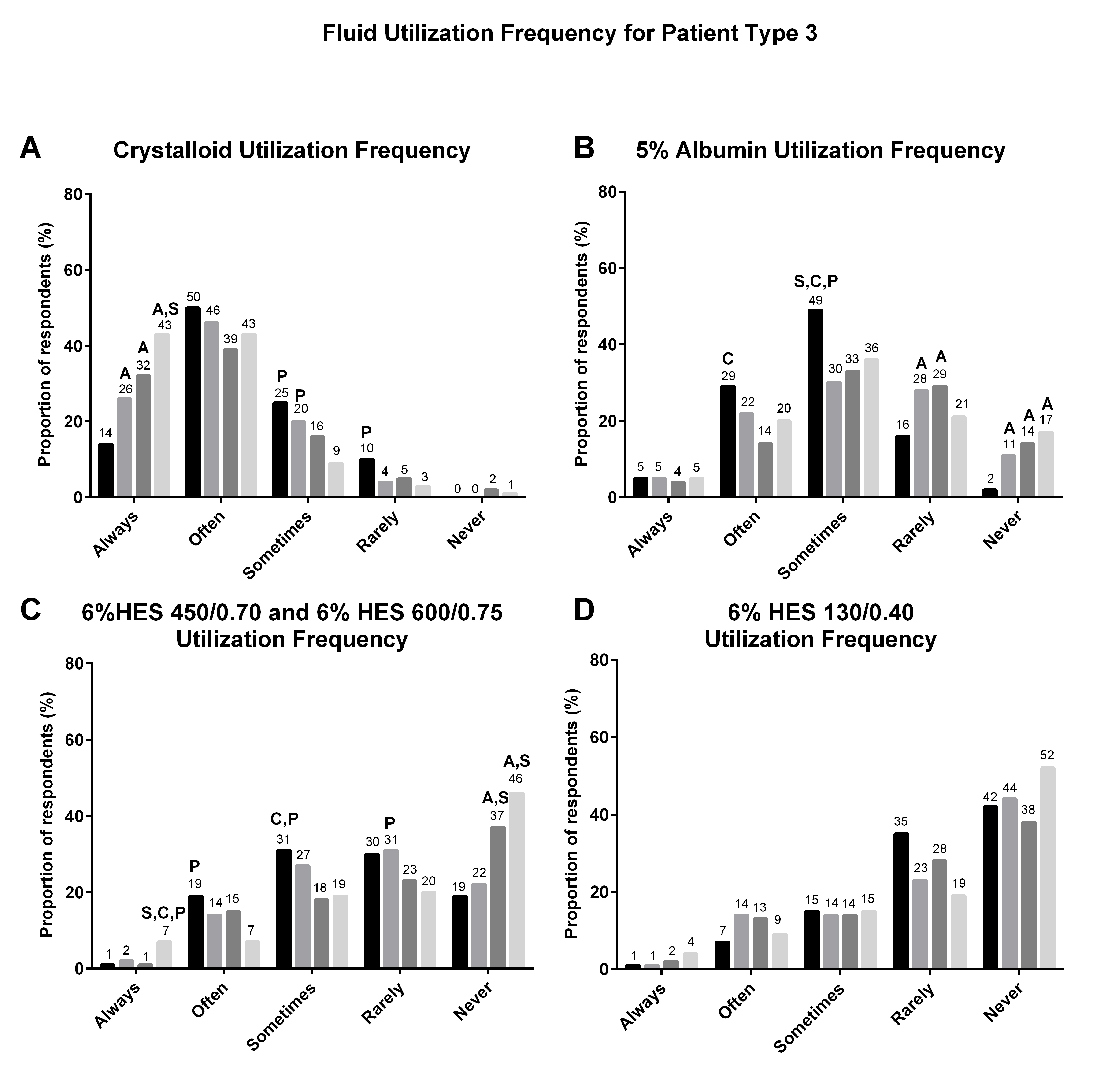

Supplement: Additional file 4: Figure S3. — Fluid choices for patient who needs volume expansion for resuscitation in sepsis (patient type 3). As follow-up questions (for “Which of the following is your first choice for a patient who needs volume expansion for resuscitation in sepsis?” As follow-up questions, the utilization frequency of (A) crystalloid, (B) 5 % albumin, (C) 6 % HES 450/0.70 AND 6 % HES 600/0.75, and (D) 6 % HES 130/0.40 was assessed by asking the question, “How often do you use each of the following or a patient who needs volume expansion for resuscitation in sepsis?” N values for panels A–D are as follows: anesthesiologists (n = 125), surgeons (n = 120), critical care medicine (n = 98), pulmonologists (n = 146). Superscripts A, S, C, and P denote differences between specialties that are statistically significant at P < 0.05. HES, hydroxyethyl starch. (JPG 1701 kb) [file 13741_2016_35_MOESM4_ESM.jpg]
